# Chronic heat stress delays immune system development and alters serotonin signaling in pre-weaned dairy calves

**DOI:** 10.1371/journal.pone.0252474

**Published:** 2021-06-04

**Authors:** Marcela G. Marrero, Bethany Dado-Senn, Sena L. Field, Guan Yang, John P. Driver, Jimena Laporta

**Affiliations:** 1 Department of Animal Sciences, University of Florida, Gainesville, Florida, United States of America; 2 Department of Animal and Dairy Sciences, University of Wisconsin, Madison, Wisconsin, United States of America; University of Illinois, UNITED STATES

## Abstract

Exposure to heat stress can alter the development and immune system function in dairy calves. Serotonin is an immunomodulatory biogenic amine that functions as a neurotransmitter and as a stress-response mediator. Our objectives were to characterize the patterns of serum serotonin concentrations and the pattern of serotonin-related genes expressed by immune cells of calves exposed to chronic heat stress or heat stress abatement during early life, and to explore whether these might relate to immune system development. Dairy calves were exposed to chronic heat stress (HS; n = 6) or heat stress abatement (cooling, CL; n = 6) across the prenatal (late gestation, last 46 d) and postnatal (from birth to weaning, 56 d) developmental windows. Blood samples were collected to harvest serum (weekly, from d 1 to 49), to isolate of circulating leukocyte mRNA (at 1, 21 and 42 d of age) and characterize immune cell populations by flow cytometry (at 21 and 47 d of age). Calves exposed to chronic heat stress pre- and postnatally had lower red blood cell counts and lower circulating serotonin, immunoglobulin G, and B-lymphocytes compared to CL calves. Circulating blood leukocyte mRNA expression of serotonin receptors -*1A*, *-1F*, *-4* and *-5* was greater, while heat shock protein 70 and immune-related genes (i.e., *TBX21*, *TLR4*, and *TGFβ*) were lower in HS relative to CL calves. Peripheral blood leukocytes from all calves secreted serotonin and interleukin-6 after *in-vitro* lipopolysaccharide stimulation. However, the HS calves produced more serotonin and less interleukin-6 than CL calves when activated *in-vitro*. Together, our data suggest that providing heat stress abatement to dairy calves across prenatal and postnatal developmental windows might modulate the serotonin synthesis pathway in ways that may benefit humoral immunity against microbial pathogens.

## Introduction

Dairy calves in hot regions can experience heat stress, both during pre- and postnatal developmental windows, when there is a lack of heat stress abatement infrastructure (i.e., sprinklers, fans, or shade) [1–3]. The immune system in dairy calves starts developing prenatally (i.e., in-utero) and reaches maturity near weaning [4]. Ingestion of colostral immunoglobulins (Ig) at birth is essential, as it enables the neonate to neutralize microbial pathogens [5]. However, calves exposed to prenatal heat stress have impaired passive transfer of Ig from ingested colostrum [3]. Colostrum-acquired IgG concentrations gradually decrease during the calf’s first month of life [6], while the calf begins to produce its own T and B-lymphocytes capable of inducing active immunity to pathogens. This process may be impaired in HS calves as it has been reported that such calves have altered immune responses that could have long-lived effects on host immunity and disease resistance [3].

Serotonin (5-hydroxytryptamine, **5-HT**) is a biogenic amine that has been shown to exert immunomodulatory effects on both the innate and adaptive immune systems [7–9]. Peripheral serotonin is synthesized in cells expressing the rate-limiting enzyme tryptophan hydroxylase (**TPH1**) that converts L-tryptophan to 5-hydroxytryptophan, which is subsequently converted to 5-HT. Serotonin signals through 14 receptor subtypes among seven receptor families [10] and various subtypes have been identified in bovine, human, and rodent immune cells [11–15]. Serotonin has been shown to recruit neutrophils to inflammation sites and to upregulate the expression of costimulatory molecules and cytokine by monocytes [7, 16]. Circulating 5-HT can also be taken up, stored, and transported by dendritic cells and platelets expressing the serotonin transporter (**SERT**) [17, 18]. In mice, activated dendritic cells release 5-HT through exocytosis during antigen presentation to enhance T-cell proliferation [17, 18]. In fact, 5-HT deficient rats have decreased proliferative T-cell responses during early life [17, 19]. Serotonin can also increase mitogen-stimulated B-cell proliferation through the activation of the 5-HT1A receptor [20]. Recently, we reported that blood leukocytes’ expression of 5-HT receptors and cytokines is increased by oral supplementation of 5-hydroxytryptophan to dairy calves [11].

Serotonin has also been extensively studied for its role in adaptive responses to physical, metabolic, and environmental stressors, primarily in humans and rodents [21–27]. For instance, rodents exposed to acute heat stress have increased 5-HT concentrations in the brain [24] and in peripheral circulation [23], suggesting that 5-HT plays a role in thermoregulation. Prolonged stress exposure, due to light cycle alterations and temperature fluctuations, induce apoptosis of 5-HT-expressing neurons in the dorsal raphe in rodents [28]. Furthermore, the hypothalamus of chronically stressed rats produces less 5-HT, which causes long-lasting thermoregulatory dysfunction [29, 30]. Serotonin can activate heat shock factor 1 (i.e., **HSF-1**), the transcriptional activator of heat shock proteins [31, 32], during heat stress exposure to prevent protein aggregation and ensure protein folding [33, 34]. However, alterations in the peripheral serotonergic system in response to prolonged heat stress have not been investigated in the bovine.

Given that 5-HT acts as an immunomodulator and as a stress-response mediator, our objective was to characterize the serotonergic-immune axis after prolonged exposure to heat stress or heat stress abatement during late gestation and early postnatal phases in dairy calves. We hypothesized that chronic heat stress would dysregulate peripheral serotonin signaling and hinder the development of humoral immune responses in dairy calves.

## Materials and methods

### Experimental design and treatments

All experimental procedures performed were approved by the University of Florida Institutional Animal Care and Use Committee (Protocol #201810202). A comprehensive description of the experimental design, housing and treatments are detailed in Dado-Senn and colleagues [1]. These authors explored Holstein calf physiological and thermoregulatory responses to similar or dissimilar thermal environments experienced during pre- and postnatal life. Herein we describe a secondary objective to examine the serotonergic-immune axis in calves under chronic heat stress or continuous heat stress abatement across both pre- and postnatal periods. Briefly, calves were subjected to either heat stress (**HS**, n = 6) or heat stress abatement (cooling, **CL**, n = 6) across the pre- and postnatal periods, for a total of 102 d (last ~46 d prenatal and 56 d postnatal). The CL dams were provided evaporative cooling through a shaded free-stall open-sided barn equipped with fans and soakers, whereas HS dams were provided with a free-stall barn without fans and soakers. Calves gestated by these dams experienced the treatments through the intrauterine environment. The temperature-humidity index (**THI**) was above 68 for the duration of the prenatal period. Each dam’s respiration rate (inhalations/min) was measured thrice weekly for 6 weeks at 1300 h, and vaginal temperature (I-button DS1922-F5#, accuracy ± 0.065°C) was recorded daily at 10 min intervals during weeks 6, 4, and 2 before calving.

**Fig 1:**
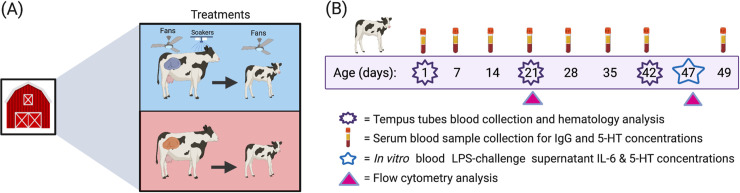
Overview of experimental design, timeline, and sampling schedule. (**A**) Dairy calves were gestated by dams exposed to heat stress (access to the shade of a free-stall barn, indicated by the red rectangle) or heat stress abatement (access to shade plus fans and water soakers, indicated by the blue rectangle) during late-gestation (~last 46 d, June-August 2018). After birth, calves were raised under similar environmental conditions to their dams until weaning (56 d of age), resulting in two treatments: heat stress (**HS**, n = 6) or cooling (**CL,** n = 6) across the pre- and postnatal periods (~ 102 d). (**B**) Blood was collected weekly from d 1 to d 49 to assess serotonin and IgG concentrations. On d 1, 21, and 42, blood was collected using Tempus RNA tubes for gene expression analysis and tubes with K_2_EDTA anticoagulant for blood hematology analysis. On d 21 and 47, blood was collected using sodium heparin tubes for flow cytometric analyses, and an on d 47 to perform an *in vitro* LPS-stimulation assay.

After birth, the calves were fed pooled colostrum from thermoneutral dams (not enrolled in our research) and housed in sand-bedded group automatic feeder pens under the shade of an open-sided barn with or without access to fans until weaning. Average daily THI remained above 68 for the duration of the postnatal period. Postnatal heat stress abatement was achieved by two calf level barrel fans to achieve airspeed of 2 m/s (VMK42-2-O 107 cm barrel fan; Schaefer, Eau Claire, WI). The sex of the calves was as follows: CL = 3 bulls, 3 heifers, and HS = 2 bulls, 4 heifers. The calves were allotted 10 L/d of milk replacer (UF Special 28% Protein/15% Fat, Bova Diflubenzuron Medicated; Southeast Milk, FL) via automatic feeders (DeLaval CF1000X, DeLaval). Weaning began at 42 d of age according to a 10-d step-down program [35]. Starter grain concentrate (Ampli-Calf Starter 20% CP Warm Weather; Purina Animal Nutrition LLC, Shoreview, MN) was provided starting at 0.2 kg/d and ending at 3 kg/d. Respiration rate (inhalations/min) and rectal temperature were measured daily at 1300 h. A schematic representation of the timeline can be found in Fig 1.

### Blood sample collection

All blood samples were collected via jugular venipuncture. Serum blood samples were collected at postnatal d 1 (24 ± 1 h of age, after colostrum feeding), 7, 14, 21, 28, 35, 42 and 49 (between 0900 and 1000 h) into 10 mL evacuated serum collection tubes (BD #366430; Franklin Lakes, NJ). Blood was allowed to clot for 20 min at room temperature and centrifuged at 3,000 × g for 20 min to harvest the serum fraction, which was aliquoted and stored at -20°C until analysis. Whole blood (3 mL) for the analysis of circulating leukocyte mRNA was collected on postnatal d 1, 21, and 42 into Tempus™ Blood RNA Tubes (#4342792, Applied Biosystems, Foster City, CA) containing 6 mL of stabilizing reagent to lyse blood cells, inactivate cellular RNases, and selectively precipitate and stabilize RNA. Upon collection, Tempus™ tubes were shaken vigorously for 30 s, placed on ice, transported to the laboratory, and stored at -20°C until the RNA extraction of peripheral leukocytes was performed. Blood samples were collected for complete blood count analyses on d 1, 21 and 42 using 5 mL tubes containing K_2_EDTA anticoagulant (BD #368047; Franklin Lakes, NJ) and immediately placed on ice. Blood was also collected using sodium heparin tubes (BD #366664; Franklin Lakes, NJ) on d 21 and 47 for flow cytometry and the lipopolysaccharide (**LPS**) stimulation assay.

### Complete blood count

An Idexx ProCyte Dx analyzer (Idexx Laboratories Inc., Westbrook, ME) was used to analyze whole blood within 2 h of collection for the concentrations of platelets, hemoglobin (**HGB**), red blood cells (**RBC)**, and white blood cells, including neutrophils, monocytes, eosinophils, and lymphocytes.

### Colostrum, apparent efficiency of absorption and serum immunoglobulin concentrations

Total IgG concentrations in pooled colostrum fed to calves (n = 4 to 6 per treatment) and individual calf serum samples (n = 6 calves per treatment; weekly from d 1 to d 49) were measured by radial immunodiffusion assay (Bovine IgG Test Kit, Triple J Farms, Bellingham, WA). Samples were diluted in 0.9% saline (serum 1:2, colostrum 1:5) so that their concentration fell within the limits of the standard curve of the assay. Five μL of standards and diluted samples were pipetted into plates containing anti-bovine IgG antibody in agarose gel and incubated in the dark at room temperature for 25 h. The precipitin ring diameter was measured in triplicates using a 7x scale loupe (# 1975; Peak Optics, GWJ Co., La Quinta, CA). The total IgG concentration was calculated based on the linear relationship between the ring diameter squared and the total IgG concentration. The inter-assay CV was 6.6% for serum and 1.1% for colostrum. The apparent efficiency of absorption (**AEA**) was calculated as in by Monteiro and colleagues [36].

### Serum serotonin concentration

Serum serotonin concentrations (n = 6 calves per treatment; weekly from d 1 to d 49) were measured using an immunoassay kit (#IM1749, Immunotech, Beckman Coulter, Marseille Cedex 9, France), according to manufacturer’s instructions. Serum samples were diluted 1:100 to fall within the range of the standard curve of the assay. All samples were assayed in duplicate. Serotonin concentrations were determined using a semi-logarithmic curve fit. The intra and inter-assay CV for all serotonin assays were 5.5 and 5.3%, respectively.

### Gene expression analysis

Total RNA was extracted from whole blood using the Tempus™ Spin RNA Isolation Kit (#4380204, Thermo Fisher Scientific, USA) as per the manufacturer’s instructions. The concentration and quality of the extracted mRNA were quantified using a Nanodrop™ (#ND-2000, Thermo Scientific, USA). All samples had an A260/280 ratio > 1.9. Extracted RNA (0.5 μg) was reverse transcribed to cDNA with iScript Reverse Transcription Supermix (#1708841, Bio-Rad, Hercules, CA) and diluted (1:3) in UltraPure™ DNase/RNase-free distilled water (#10977015, Thermo Fisher, USA). Quantitative real-time PCR was conducted using the Bio-Rad CFX96 Touch Real-Time PCR Detection System (#1855195). Reaction mixtures with a final volume of 10 μL per well contained 6.25 μL of SSoFast™ EvaGreen Supermix (#1725203, Bio-Rad), 0.5 μL of each forward and reverse primer, 0.75 μL of UltraPure™ distilled water and 2 μL of cDNA. The PCR cycle conditions were as follows: 1 cycle for 3 min at 95°C then 50 cycles of 10 s at 95°C and 30 s at 60°C followed by melt curve (65°C to 95°C in 0.5°C increments for 5 s). All plates included a positive (cDNA pooled sample) and a negative control (RNA-free water). All samples were assayed in duplicate.

The geometric mean of two housekeeping genes (*RSP9*, *β-actin*) was used to calculate normalized gene expression (ΔCt) for statistical analysis. After statistical analysis, the estimates (ΔΔCt) were used to calculate the fold change according to Livak and Schmittgen [37]. Gene expression is reported as log_2_(fold change, FC). We evaluated the gene expression of heat shock factor 1 (*HSF1*), heat shock proteins (*HSP70*, *HSP72* and *HSP90*), T-cell transcription factors (*GATA3*, *FoxP3*, *TBX21* and *RORγδ*), toll like receptors 2 and 4 (*TLR2* and *TLR4*), L-selectin (*CD62L*), co-stimulatory molecules (*CD28* and *CD80*), interleukins (*IL2*, *IL4*, *IL10* and *IL12B*), interleukin 2 receptor (*CD25*), transforming growth factor beta (*TGFB1*), interferon gamma (*IFNG*), apoptosis regulator BAX (*BAX*), Bcl-2-related protein A1 (*BCL2A1*) and serotonin machinery related genes including *SERT*, *TPH1* and serotonin receptors (*5*-*HT1A*, -*1B*, -*1D*, -*1F*, -*2A*, -*2B*, -*2C*, *-4*, -5, -*6*, -*7*). A six-point two-fold dilution cDNA standard curve was used to validate the primers. Primers were validated if the efficiency was between 90 and 105%, the R^2^ ≥ 0.90, and the primer pair displayed melting curves with a single peak indicating a pure, single amplicon. Primer sequences and sources can be found in **S1 Table**.

### Ex-vivo whole blood LPS stimulation

Interleukin 6 (**IL-6**) and 5-HT production were measured from the cell culture supernatant of *in vitro* LPS-stimulated whole blood collected and cultured at d 47 of age. Five-hundred μL of whole blood in 2 mL of Gibco RPMI 1640 medium (#11-875-085, Invitrogen) containing 1% of antibiotic/antimycotic (#15-240-062, Invitrogen, CA) were plated in 24 well tissue culture plates with increasing doses of LPS (0, 0.5, 1, and 5 μg/mL, L6136, Serratia marcescens, Sigma Aldrich) and incubated at 37°C and 5% CO_2_ for 24 h. Afterwards, plates were centrifuged to collect the cell culture supernatants, which were analyzed using ELISAs against IL-6 (DY8190, R&D Systems, Inc., MN, USA) and 5-HT (IM1749, Immunotech, Beckman Coulter, Marseille Cedex 9, France) according to the manufacturer’s directions. The intra-assay CVs were 15.1% and 5.8% for 5-HT and IL-6, respectively.

### Flow cytometry analysis

Whole blood (100 μL) was transferred from the sodium heparin treated tubes to round bottomed FACS tubes containing 2 mL of PBS and centrifuged at 700 g for 3 min at 4°C. The resulting cell pellet was treated with an ammonium chloride-based lysis buffer to remove red blood cells, after which the cells were washed with 2 mL of PBS 1X, centrifuged, and the supernatant discarded. The ammonium chloride-based buffer step was repeated, and pelleted cells were re-washed with 2 mL of PBS. To block Fc receptor binding, cells were incubated with 10 μL rat IgG (#I4131, Sigma-Aldrich, Saint Louis, MO) for 10 min at 4°C. Cells were then stained for 30 minutes at 4°C with the following fluorochrome-conjugated antibodies: anti-CD62L (BAQ92A, Alexa Fluor 647, #WS0515B-100, Kingfisher, US), anti-CD44 (IL-A118, FITC, #MCA2433F, Bio-Rad, US), anti-CD4 (CC30, PerCP, #MCA834GA, Bio-Rad, US), anti-CD8 (CC63, RPE, #MCA837PE, Bio-Rad, US), anti-CD21 (CC51, FITC, #MCA5953F, Bio-Rad, US), anti-TCRγδ (GB21A, Alexa Fluor 647, #WSC0578B-100, Kingfisher, US), and anti-CD14 (TÜK4, PE, #MCA1568PE, Bio-Rad, US) antibodies. Alexa647 (#A20186, ThermoFisher Sci., USA) and PerCP (#LNK07, Bio-Rad, U.S.A) fluorochrome conjugation kits were used to conjugate anti-CD4 (CC30) and anti-TCRγδ (GB21A), which were purchased unconjugated. Propidium iodide was used for the exclusion of dead cells during analysis. Stained cells were washed once, resuspended in 500 μL of PBS, and acquired with an Accuri C6 flow cytometer. All flow cytometry data were analyzed using FlowJo software (version 10.7.1, Treestar, Palo Alto, CA) using the gating strategies described in **S1 Fig**.

### Statistical analysis

Data were analyzed using R version 3.5.1 (R Foundation for Statistical Computing; Vienna, Austria). A priori statistical power analysis was performed. Six animals per treatment were needed to detect gene expression differences of two or more cycle thresholds (significance < 0.05, 90% power). Mixed models were used to analyze variables with repeated measures such as peripheral blood leukocyte gene expression (ΔCt), serum IgG and 5-HT concentrations, flow cytometry (% and mean fluorescence intensity, **MFI**), and hematology analysis. The model included treatment, day, and their interaction as fixed effects, ID as a random effect, and the autoregression (CorAR1) as the covariance structure. Interleukin-6 and 5-HT concentrations in cell culture supernatant after *in vitro* blood LPS-stimulation were log-transformed to ensure normality and re-analyzed using a linear model including treatment, concentration, and their interaction as fixed effects. Homogeneity of variance and normality of residuals were evaluated by plotting residuals *vs*. fitted values, and influential points were detected using Cook’s distance test. Statistical significance was declared at *P* ≤ 0.05 and tendencies at 0.05 < *P* ≤ 0.10.

## Results

### Physiological parameters

**Fig 2:**
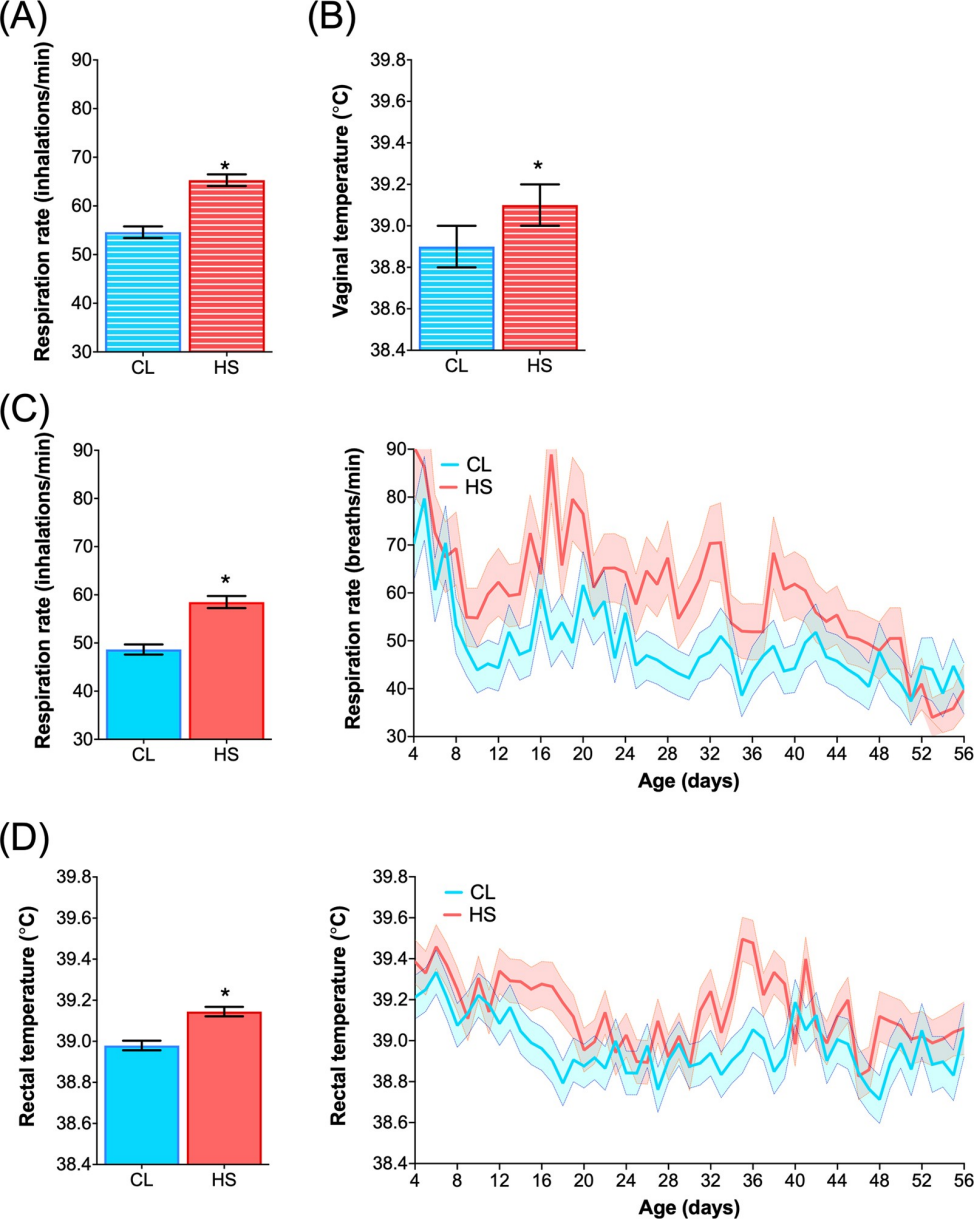
Respiration rate and body temperature in dams and dairy calves. Dam’s (**A**) respiration rate (inhalations/min) recorded twice a week for 6 weeks prior to calving, and (**B**) vaginal temperature recorded every 10-min during weeks 6, 4 and 2 prior to calving illustrated as the LSM of the treatment. Dairy calves’ (**C**) respiration rate and (**D**) rectal temperature measured daily at 1300 h across the pre-weaning period (56 d) illustrated as the LSM of the treatment (left-graph) and as the LSMs of the treatment by day interaction (right-graph). Dairy calves were exposed to heat stress (HS; n = 6) or heat stress abatement (CL; n = 6) across pre- and postnatal phases (late gestation and pre-weaning) for a total of 102 d. Blue and red bars with horizontal white lines denote CL dams (prenatal CL exposure) and HS dams (prenatal HS exposure), respectively, whereas blue and red bars denote dairy calves’ postnatal CL and HS exposure, respectively. No treatment by day interaction effects were found between dam’s or calves respiration rate or body temperatures (*P* > 0.15). (*) indicate significance (*P* ≤ 0.05). Data was adapted from Dado-Senn and colleagues, 2020 [1].

Respiration rates and core body temperatures were recorded from the dam and offspring to determine their physiological responses to heat stress and heat stress abatement [1]. Dams receiving heat stress abatement had significantly lower respiration rates and vaginal temperatures compared with heat stressed dams (54.6 *vs*. 65.3 ± 1.2 bpm, and 38.9 *vs*. 39.1 ± 0.1°C, respectively, both *P* < 0.01; Fig 2A and 2B). Similarly, calves receiving pre- and postnatal heat stress abatement had lower respiration rates and rectal temperatures compared to calves receiving pre- and postnatal heat stress (38.98 *vs*. 39.14 ± 0.02°C, and 48.65 *vs*. 58.60 ± 1.16 bpm, both *P* < 0.01; CL *vs*. HS, respectively; Fig 2C and 2D). These results confirm that the lack of heat stress abatement increases physiological indicators of heat stress, both pre- and postnatally.

### Serum and colostrum IgG concentrations, and apparent efficiency of absorption

**Fig 3:**
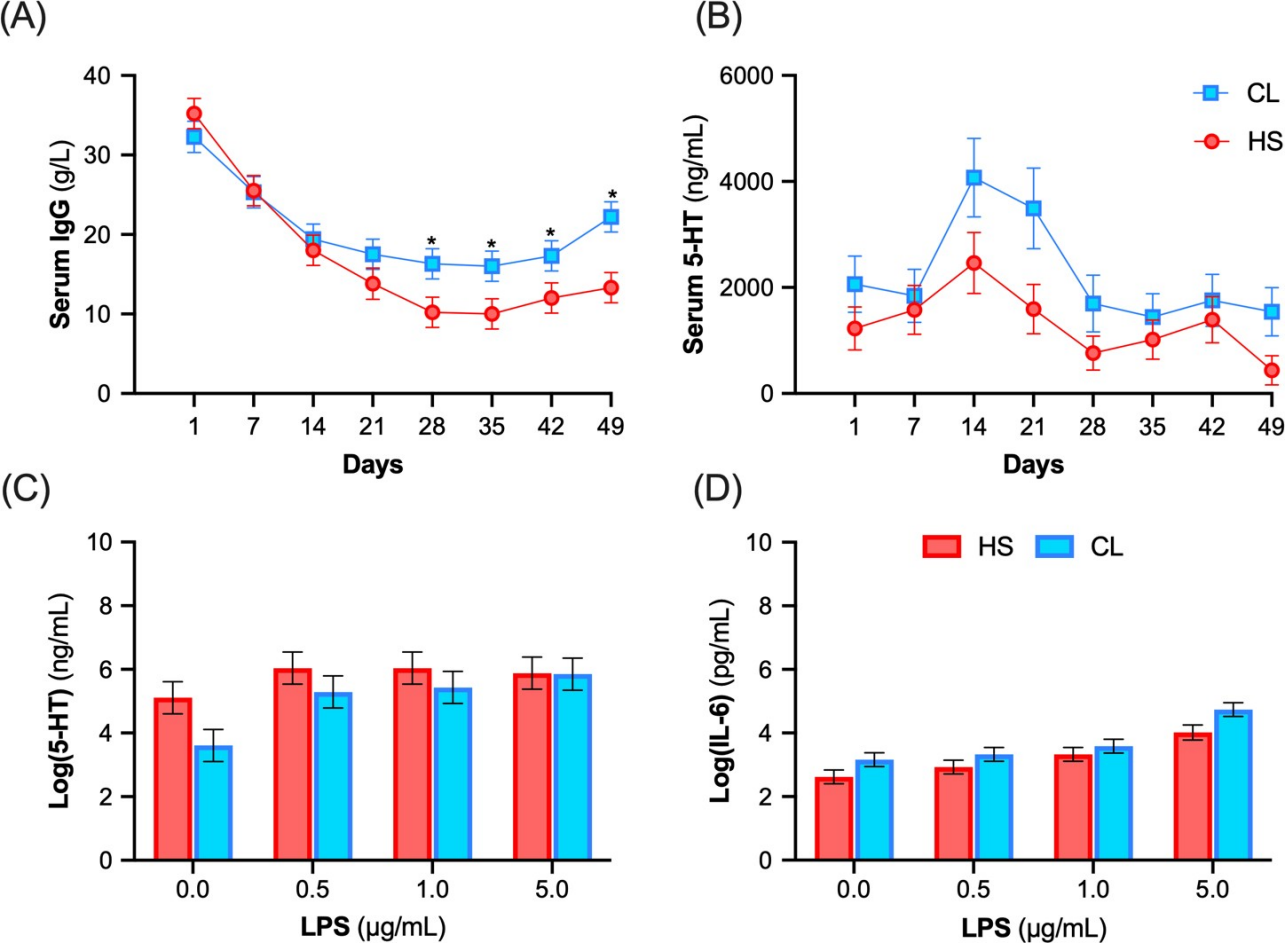
Weekly serum IgG and serotonin concentrations and whole blood production of serotonin and interleukin 6 after *in vitro* LPS-challenge. Serum (**A**) IgG and (**B**) serotonin (5-HT) concentrations of calves from d 1 to d 49 postnatal. (**C**) Log-transformed serotonin (5-HT), and (**D**) log-transformed interleukin-6 (IL-6) concentrations 24-h after *ex vivo* blood stimulation with increasing doses of lipopolysaccharide (LPS, 0, 0.5, 1 and 5 μg/mL) at postnatal d 47. Calves were exposed to heat stress (HS, shade: n = 6) or heat stress abatement (CL, shade plus fans; n = 6) across pre- and postnatal periods (late gestation and pre-weaning). Blue squares denote CL treatment, and red circles denote HS treatment. There was a treatment by day interaction for serum IgG concentrations (*P* = 0.05), but no interaction effects for serum 5-HT concentrations (*P* = 0.38), and plasma supernatant concentrations of 5-HT (*P* = 0.54) or IL-6 (*P* = 0.76). Concentrations of 5-HT and IL-6 were log-transformed for analysis due to a lack of normality. Back transformed data are presented in S2 Table. Asterisk (*) indicate significance (*P* ≤ 0.05).

To determine whether temporal differences in humoral immunity exist between HS and CL calves, IgG concentrations were measured from the colostrum fed to calves and from serum samples collected from calves weekly from d 1 to d 49. The colostrum IgG concentration (87.5 *vs*. 72.3 ± 12.5 g/L, fed to CL *vs*. HS, respectively) and AEA (40.5 *vs*. 39.8 ± 10.9%) did not differ between treatments (*P* > 0.43). However, there was a treatment by day interaction for serum IgG concentrations, whereby CL calves had greater IgG concentrations from 28 to 49 days of age compared to HS calves (*P* = 0.05; Fig 3A).

### Serum serotonin concentrations

To assess the effect of chronic heat stress and heat stress abatement on circulating 5-HT levels, serum 5-HT concentrations were assessed weekly from d 1 to 49. There was a treatment effect, whereby CL calves had greater serum 5-HT concentrations compared to HS calves during the preweaning period (*P* = 0.04; Fig 3B).

### Complete blood counts

Complete blood counts (i.e., hematology parameters and white blood cell differentials) were assessed on d 1, 21, and 42. There was a treatment effect for the number of RBC, HGB concentration, and the hematocrit (**HCT**) percentage, whereby CL calves had higher RBC, HGB, and HCT relative to HS calves (*P* < 0.02; Table 1). There was a treatment by day interaction for eosinophil counts, whereby CL calves had lower eosinophil counts on d 1 relative to HS calves (*P* = 0.04). There was a day effect for neutrophil, monocyte, and lymphocyte counts (*P* < 0.0001; Table 1).

**Table 1:** Complete blood counts of Holstein dairy calves at 1, 21 and 42 days of age.

			Treatment^1^							
		CL			HS	*P*-value^3^				
Item	day 1^2^	day 21	day 42	day 1	day 21	day 42	Trt		day	Trt×day
Red blood cells, M/μL	7.43 ± 0.45^a,b^	8.40 ± 0.38^a,b^	9.45 ± 0.05^a^	6.95 ± 0.41^b^	6.93 ± 0.41^b^	7.83 ± 0.46^a,b^		**0.01**	**0.02**	0.36
Hemoglobin, g/DL	9.71 ± 0.59^a^	9.59 ± 0.43^a^	10.2 ± 0.52^a^	8.75 ± 0.47^a,b^	8.24 ± 0.47^b^	8.71 ± 0.52^a,b^	**0.02**		0.45	0.86
Hematocrit, %	33.4 ± 2.0^a^	30.4 ± 1.67^a^	32.4 ± 2.01^a^	29.5 ± 1.83^a,c^	24.4 ± 1.83^b^	26.0 ± 2.03^b,c^	**0.02**		**0.05**	0.75
Platelets, 10^3^/μL	353 ± 68.1^a^	637 ± 57.3^b^	522 ± 68.1^b^	298 ± 61.1^a^	557 ± 62.7^b^	561 ± 67^b^	0.28		**0.001**	0.54
White blood cells, 10^3^/μL	8.62 ± 1.31^a^	9.76 ± 1.19^a^	8.82 ± 1.32^a^	10.76 ± 1.19^a^	8.88 ± 1.19^a^	9.96 ± 1.32^a^	0.48		0.88	0.31
Neutrophils, 10^3^/μL	6.21 ± 1.14^a,b^	4.44 ± 0.92^a,c^	2.91 ± 1.11^c,d^	7.87 ± 1.03^b^	2.88 ± 1.00^c,d^	3.75 ± 1.12^a,c^	0.59		**0.01**	0.21
Lymphocytes, 10^3^/μL	2.95 ± 0.54^a,b^	5.38 ± 0.05^c^	5.19 ± 0.45^c^	2.82 ± 0.54^a^	5.05 ± 0.54 ^c^	4.35 ± 0.57^b,c^	0.3		**<0.0001**	0.57
Monocytes, 10^3^/μL	0.00 ± 0.20^a^	1.06 ± 0.18^b,c^	1.49 ± 0.20^b,d^	0.00 ± 0.18^a^	0.84 ± 0.19^c^	1.75 ± 0.19^d^	0.41		**<0.0001**	0.11
Eosinophils, 10^3^/μL	0.03 ± 0.01^a^	0.01 ± 0.01^a^	0.05 ± 0.01^a^	0.09 ± 0.01^b^	0.03 ± 0.01^a^	0.04 ± 0.01^a^	**0.04**		**0.005**	**0.04**

^1^ Calves were exposed to heat stress (HS; n = 6) or heat stress abatement (CL; n = 6) treatments (Trt) across pre- and postnatal periods (late gestation and pre-weaning) for a total of 102 d.

^2^ Blood samples collected on d 1, 21 and 42 postnatal. Data are presented as LSM ± SEM of the interaction treatment (Trt) × day.

^3^ Bolded *P-*values indicate significant differences (*P* ≤ 0.05) and letters (a-d) indicate significant differences between treatments across different time points.

### Peripheral blood leukocyte mRNA gene expression

Peripheral blood leukocyte heat stress-related gene expression was assessed on d 1, 21, and 42. Calves provided heat stress abatement had greater *HSP70* mRNA expression when compared to HS calves (*P* = 0.02; Table 2). There was also a day effect for *HSP90* mRNA expression (*P* = 0.01) which was greater at d 0 compared to d 42 in both treatments (*P* = 0.01; Table 2). No differences were found for *HSF1* and *HSP72* mRNA expression (*P* > 0.13; Table 2). From the genes involved in 5-HT synthesis and metabolism, there was a treatment by day interaction for *TPHI* mRNA expression (*P* = 0.01) where CL calves had lower mRNA expression at d 1 compared to HS calves (*P* < 0.01). No differences in *SERT* mRNA expression were found across the preweaning period (*P* > 0.13; Table 2).

**Table 2:** mRNA expression of peripheral blood leukocytes isolated from dairy calves at 1, 21 and 42 days of age.

Gene	mRNA expression of CL relative to HS calves^a^					
	day 1	day 21	day 42	*P*-value^c^		
	Log_2_FC^b^ ± SEM	Log_2_FC ± SEM	Log_2_FC ± SEM	Trt	day	Trt×day
*HSF1*	0.12 ± 0.35	0.70 ± 0.35	0.04 ± 0.36	0.14	0.32	0.44
*HSP70*	0.57 ± 0.17	0.73 ± 0.12	0.58 ± 0.10	**0.02**	0.56	0.29
*HSP72*	-0.22 ± 0.27	-0.19 ± 0.27	-0.12 ± 0.27	0.24	0.33	0.94
*HSP90*	-0.13 ± 0.26	-0.36 ± 0.28	0.13 ± 0.27	0.36	**0.01**	0.42
*RORgd*	-0.63 ± 1.84	-1.83 ± 1.89	0.94 ± 1.86	0.58	**0.01**	0.45
*FoxP3*	0.90 ± 0.35	0.40 ± 0.35	0.17 ± 0.36	0.10	**< .0001**	0.33
*GATA3*	0.34 ± 0.33	0.08 ± 0.33	-0.16 ± 0.34	0.86	**< .0001**	0.54
*TBX21*	0.64 ± 0.44	0.72 ± 0.45	0.72 ± 0.45	**0.05**	**< .0001**	0.98
*CD62L*	-0.65 ± 0.46	0.80 ± 0.53	-0.47 ± 0.49	0.68	**0.04**	0.24
*CD25*	-0.35 ± 0.49	0.47 ± 0.53	-0.20 ± 0.53	0.89	**< .0001**	0.36
*CD28*	0.56 ± 0.39	0.00 ± 0.39	-0.04 ± 0.40	0.70	**< .0001**	0.43
*CD80*	0.45 ± 0.40	0.31 ± 0.40	0.13 ± 0.41	0.19	0.24	0.86
*TLR2*	0.45 ± 0.55	0.77 ± 0.57	0.17 ± 0.59	0.22	0.77	0.76
*TLR4*	0.94 ± 0.44	0.31 ± 0.45	0.32 ± 0.46	**0.02**	0.86	0.52
*BCL2A1*	-0.43 ± 0.67	-0.55 ± 0.67	0.35 ± 0.69	0.65	0.10	0.54
*BAX*	0.18 ± 0.29	0.02 ± 0.31	0.41 ± 0.29	0.48	**< .0001**	0.60
*TGFB1*	0.78 ± 0.31	0.57 ± 0.32	0.52 ± 0.32	**<0.01**	**0.03**	0.73
*IFNG*	1.10 ± 0.68	-0.06 ± 0.67	0.51 ± 0.66	0.66	**< .0001**	0.44
*TNFA*	0.13 ± 0.39	0.21 ± 0.39	0.07 ± 0.40	0.69	0.58	0.96
*IL1B*	-0.16 ± 0.60	-0.11 ± 0.63	-0.07 ± 0.65	0.72	0.28	0.99
*IL2*	0.32 ± 0.32	-0.41 ± 0.34	0.22 ± 0.32	0.15	**< .0001**	0.47
*IL4*	-1.07 ± 0.07	-1.11 ± 0.07	1.00 ± 0.07	0.21	**< .0001**	0.44
*IL10*	-0.03 ± 0.49	0.16 ± 0.51	-0.40 ± 0.53	0.85	**0.02**	0.76
*IL12B*	-0.35 ± 0.56	-0.16 ± 0.56	-0.64 ± 0.58	0.41	**<0.01**	0.80
*SERT*	0.85 ± 0.57	0.06 ± 0.58	0.37 ± 0.61	0.29	0.14	0.58
*TPH1*	-2.13 ± 0.49	-0.46 ± 0.46	0.05 ± 0.50	**<0.01**	0.67	**0.01**
*5HT1A*	-3.35 ± 0.95	-2.00 ± 0.90	-1.41 ± 1.41	**<0.01**	**< .0001**	0.35
*5HT1B*	-2.09 ± 0.50	-1.11 ± 0.47	-0.01 ± 0.49	**0.01**	0.23	**0.02**
*5HT1D*	-3.28 ± 0.53	-1.22 ± 0.51	0.06 ± 0.53	**<0.01**	**< .0001**	**<0.01**
*5HT1F*	-2.34 ± 0.56	-1.19 ± 0.56	-0.35 ± 0.58	**0.01**	**< .0001**	0.06
*5HT2A*	1.14 ± 0.59	0.01 ± 0.62	1.13 ± 0.64	**0.03**	**0.05**	0.46
*5HT2B*	-0.52 ± 0.38	0.07 ± 0.38	-0.15 ± 0.41	0.34	0.35	0.57
*5HT2C*	-3.51 ± 0.54	-1.26 ± 0.50	0.99 ± 0.52	**0.01**	**< .0001**	**< .0001**
*5HT4*	-3.02 ± 0.76	-2.02 ± 0.76	-0.77 ± 0.82	**<0.01**	0.08	0.14
*5HT5*	-2.95 ± 0.98	-2.31 ± 0.98	-0.43 ± 1.05	**0.02**	**0.01**	0.15
*5HT6*	-1.58 ± 0.43	-0.72 ± 0.45	0.56 ± 0.47	**0.05**	0.69	**0.01**
*5HT7*	-1.83 ± 0.40	-1.53 ± 0.40	0.05 ± 0.41	**<0.01**	0.60	**0.01**

^a^ Calves were exposed to heat stress (HS; n = 6) or heat stress abatement (CL; n = 6) treatments (Trt) across pre- and postnatal periods (late gestation and pre-weaning) for a total of 102 d.

^b^ Gene expression is presented as Log_2_ Fold Change (Log_2_FC) of CL relative to HS calves at d 1, 21 and 42 postnatal using the LSM ± SEM of the interaction treatment (Trt) × day.

^c^ Significance was declared at *P* ≤ 0.05 (bold) and tendencies at 0.05 < *P* ≤ 0.10.

From the 5-HT receptors, CL calves had greater *5-HT2A* (*P* = 0.03) and lower *5-HT1A*, -*1F*, -*4* and -*5* mRNA expression relative to HS calves ((*P* < 0.04; Table 2). There was also a treatment by day interaction (*P* < 0.02; S2 Fig), where CL calves had lower *5*-*HT1B*, -*1D*, -*2C*, and -*7* mRNA expression on d 1 and 21 (*P* < 0.04) compared to HS calves. In contrast, *5-HT2C* expression also tended to be greater on CL compared to HS calves at d 42 postpartum (*P* = 0.08). Among the immune-related genes assessed, CL calves had greater *TBX21*, *TLR4*, and *TGFB1* mRNA expression (*P* < 0.05) and tended to have greater *FoxP3* mRNA expression when compared to HS calves (*P* = 0.10; Table 2). The mRNA expression of the genes that had a significant treatment by day interaction can be visualized in S2 Fig.

### Ex-vivo LPS stimulation of whole blood

Whole blood from CL and HS calves collected on postanal d 47 was incubated *in vitro* with increasing concentrations of LPS for 24 h. There was a treatment effect for 5-HT, whereby whole blood from CL calves secreted less 5-HT compared to HS calves (*P* = 0.04). There was an LPS-treatment effect (*P* = 0.02) whereby administration of any concentration of LPS increased culture supernatant 5-HT concentrations compared to unstimulated cells (5.66, 5.74, and 5.87 *vs*. 4.36 ± 0.36 μg/mL log(5-HT), for 0.5, 1 and 5 *vs*. 0 μg/mL of LPS, respectively, *P* < 0.01; Fig 3C). No interaction between calf treatment and LPS dose was detected.

Blood from CL calves tended to secrete more IL-6 compared to HS calves (*P* = 0.08; Fig 3D). Concentrations of IL-6 in culture supernatant were higher at the 1 or 5 μg/mL doses of LPS compared with unstimulated cells (3.46 and 4.38 *vs*. 2.89 ± 0.15 μg/mL log(IL-6), respectively; *P* < 0.01). No interaction between calf treatment and LPS dose was detected for IL-6 concentrations (*P* > 0.76).

### Flow cytometric analyses

**Fig 4:**
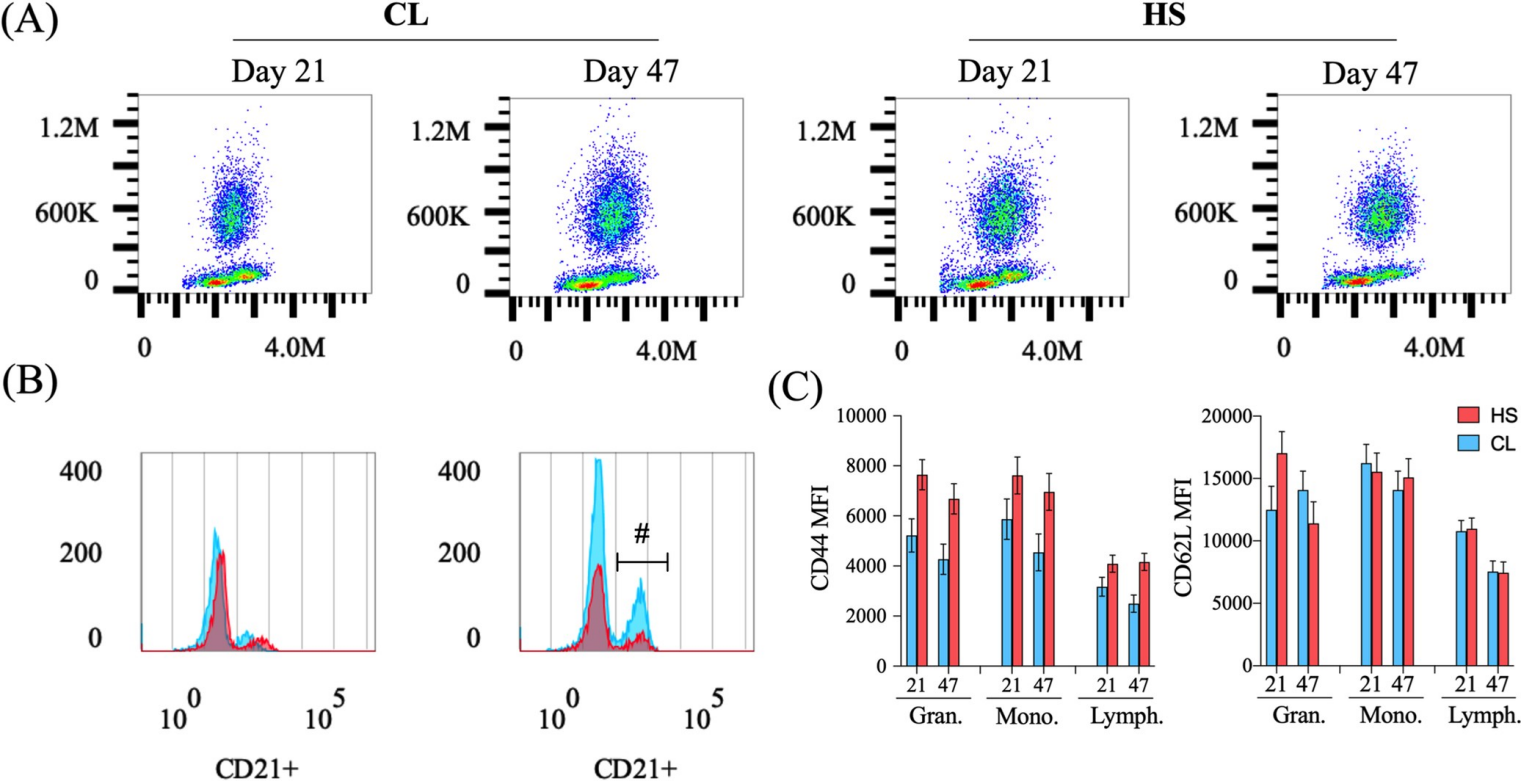
Flow cytometric analysis of peripheral blood leukocyte populations on postpartum d 21 and 47. (**A**) Forward versus side scatter plots displaying granulocyte, monocyte, and lymphocyte populations in dairy calves’ peripheral leukocytes, (**B**) lymphocyte CD21+ populations (B-cells) on d 21 (left plot), and d 47 (right plot) in dairy calves, and (**C**) CD44 (bottom left plot) and CD62L (bottom right plot) mean fluorescence intensity (MFI) of granulocytes, monocytes and lymphocytes per treatment in dairy calves at 21 and 47 d of age. Dairy calves were exposed to heat stress (HS; n = 6) or heat stress abatement (CL; n = 6) across pre- and postnatal phases (late gestation and pre-weaning) for a total of 102 d. Blue color denotes CL treatment, and red denotes HS treatment. There was a treatment by day interaction for CD21+ B-cells (*P* = 0.02) but no interactions were found for the CD62L or CD44 expression of granulocytes, lymphocytes or monocytes (*P >* 0.07). Pound sign (#) indicate tendency (0.05 < *P* ≤ 0.10).

Peripheral blood leukocytes were analyzed by flow cytometry at postnatal d 21 and 47. The proportion of lymphocytes and granulocytes among leukocytes was similar between CL and HS calves (*P* > 0.22, Fig 4A). There was a day effect for monocyte level, whereby both treatments had a lower percentage of monocytes among live cells at postnatal d 47 compared to d 21 (*P* = 0.03; Table 3). There was also a treatment by day interaction for CD21+ B-cells as a percentage of lymphocytes, which respectively increased by 7.1% in the CL calves and 3.2% in the HS calves from d 21 to 47 postnatal (*P* = 0.02; Table 3). This resulted in 2.3-fold greater B-cell percentage in CL calves compared to HS calves. The percentage of TCRγδ+ T-cells as a proportion of lymphocytes was affected by day, whereby the TCRγδ+ percentage decrease at d 47 compared to d 21 in both treatments (*P* = 0.05; Table 3). No differences in the proportion of CD4+ and CD8+ positive lymphocytes were found between HS and CL calves (*P* > 0.20, Table 3).

**Table 3:** Flow cytometric analysis of peripheral blood leukocytes of CL and HS calves at 21 and 47 d of age.

		Treatment^a^					
	CL		HS		*P-*value^c^		
Cell population	day 21^b^	day 47	day 21	day 47	Trt	day	Trt×day
Granulocytes, %	37.4 ± 4.21	43.4 ± 3.99	37.4 ± 3.99	38.3 ± 3.99	0.58	0.22	0.33
Monocytes, %	16.1 ± 2.06	10.5 ± 2.06	17.9 ± 2.06	13.7 ± 2.06	0.27	**0.03**	0.72
Lymphocytes, %	29 ± 3.48	36.5 ± 3.48	33.8 ± 3.48	32.5 ± 3.48	0.92	0.39	0.24
CD4, %	7.6 ± 1.18	5.5 ± 1.18	5.5 ± 1.08	5.8 ± 5.08	0.46	0.48	0.29
CD8, %	12.3 ± 2.05	14.4 ± 2.05	16.6 ± 1.87	17.0 ± 1.87	0.2	0.35	0.48
CD21, %	11.1 ± 2.03	18.2 ± 2.03	9.8 ± 2.03	12.9 ± 2.03	0.27	**<0.001**	**0.02**
TCRγδ, %	40.6 ± 3.43	34.6 ± 3.43	35.6 ± 3.43	33.4 ± 3.43	0.5	0.05	0.32

^a^ Calves were exposed to heat stress (HS; n = 6) or heat stress abatement (CL; n = 6) treatments (Trt) across pre- and postnatal periods (late gestation and pre-weaning) for a total of 102 d.

^b^ Data are presented as LSM ± SEM of the interaction treatment (Trt) × day.

^c^ Bolded *P*-values indicate significant differences at *P* ≤ 0.05 (bold) and tendencies at 0.05 < *P* ≤ 0.10.

The MFI of anti-CD44 antibody staining on granulocytes, monocytes, and lymphocytes was lower in CL calves relative to HS calves (*P* < 0.02, Fig 4C). There was a tendency for a treatment by day interaction for CD62L MFI on granulocytes (*P* = 0.07), whereby HS calves had lower CD62L MFI expression at d 47 compared with d 21 (*P* = 0.04; Fig 4C). No differences in granulocyte CD62L expression by day was found in the CL calves (*P* > 0.11; Fig 4C). Lymphocyte CD62L MFI had a day effect whereby lymphocytes from both treatments had lower CD62L expression at postnatal d 47 compared with d 21 (*P* < 0.001; Fig 4C). No differences were observed between treatments for monocyte CD62L MFI (*P* > 0.16, Fig 4C).

## Discussion

Heat stress has been previously associated with impaired immune responses in dairy calves [3, 36, 38], and altered 5-HT metabolism [29, 30]. Herein, we report that dairy calves’ early life exposure to prolonged heat stress leads to lower RBCs and lower circulating serotonin, IgG concentrations, and B lymphocytes compared to calves receiving heat stress abatement. To our knowledge, this is the first report characterizing the impact of chronic heat stress on the bovine 5-HT-immune axis.

The adaptive immune system development is essential for disease resistance. Dairy calves are born with a naïve immune system which develops gradually reaching maturity at approximately five months of age [39]. During this period, calves acquire the ability to produce endogenous antibodies that protect them from infectious diseases [5]. In the current study, we observed that providing pre- and postnatal heat stress abatement did not impair AEA and that CL dairy calves increased their serum IgG concentrations starting at postnatal d 28 relative to calves exposed to chronic heat stress. A study researching the effects of solely prenatal HS or CL, reported that calves born to CL cows produced greater concentrations of postnatal serum IgG relative to calves born to HS cows [3]. Although it is possible that the lower IgG concentrations in our HS calves are arising from the prenatal hyperthermia experienced in utero, our CL calves also experienced greater increment of CD21+ B-cells during the postnatal pre-weaning period relative to HS calves. Thus, it is possible that providing heat stress abatement to calves during early developmental phases aids CD21+ B-cell proliferation and/or differentiation into immunoglobulin secreting cells during the postnatal phase.

Calves provided with pre- and postnatal heat stress abatement had greater serum 5-HT concentrations throughout the postnatal pre-weaning period relative to HS calves. It was recently demonstrated, using a *TPH1* knockout model (lacking peripheral 5-HT), that 5-HT plays an essential role in erythropoiesis and RBC survival [40]. Amireault and colleagues (2011) reported that the proliferative capacity of erythroid precursor cells is enhanced by 5-HT2A and 5-HT2B agonists, whereas mature RBCs lack 5-HT receptor expression [40]. Further, 5-HT can act as an antioxidant with similar effects as vitamin E effects in prolonging RBCs’ half-life in circulation [41]. In our experiment, higher 5-HT concentrations were accompanied by greater RBC and HGB concentrations in CL calves. Calves provided with heat stress abatement also had greater levels of HCT. This has been previously reported in *in utero* CL calves [1, 3], and in calves supplemented with a 5-HT precursor [12]. Thus, our CL calves may have had greater RBC count and HGB concentrations due to the additional 5-HT these calves produced, possibly promoting hematopoietic progenitor development and RBC survival.

No differences in WBC parameters were found between treatments, except for eosinophils, which were reduced in CL calves at d 1 postnatal. Eosinophils are granulocytes which phagocytize parasites and sometimes contribute to allergic inflammatory responses [5]. Interestingly, Strong *et al*. (2015) reported lower eosinophil counts in *in utero* HS calves [42]. The reason why this report conflicts with our findings is uncertain but may have to do with the difference that the calves in our study were also provided with heat stress or heat stress abatement postnatally. Nevertheless, the effects of eosinophil function, and the potential involvement of heat stress in eosinophil regulation is yet to be determined.

Calves under chronic heat stress had reduced *HSP70* mRNA expression in peripheral blood leukocytes when compared to CL calves. The expression of *HSP70* is induced under heat stress conditions; it functions as a chaperone protein that prevents unfolding [43] and can promote the degradation of defective proteins [24, 44]. Collier *et al*. (2006) reported that bovine mammary epithelial cells cultured under acute thermal stress had higher *HSP70* mRNA expression within the first four hours of thermal exposure which declined after a prolonged period of thermal exposure [45]. This change in *HSP70* expression was attributed to known differences in the physiological response to acute and chronic heat stress. The acute heat stress response is characterized by short-term heat stress adaptations designed to promote cellular homeostasis within seconds to minutes after the onset of heat stress. Conversely, the chronic heat stress response is characterized by long-term heat stress adaptations that include reprogramming gene expression (i.e., *HSP70*) and endocrine changes within hours or days from exposure to stressor/s to enhance efficiency of metabolism during stress [46, 47]. The implications of these reports for the current study include the possibility that *in utero* hyperthermia led to a chronic level of heat stress that promoted the reduced *HSP70* mRNA expression in our HS (relative to CL calves) in a similar way to the aforementioned finding that bovine mammary epithelial cells downregulate *HSP70* after chronic heat stress.

Calves exposed to pre- and postnatal heat stress had higher *TPH1* mRNA expression in peripheral blood leukocytes, which was accompanied by lower serum 5-HT concentrations. The lower serum 5-HT concentrations in HS calves relative to CL calves may have been the downstream consequence of the increased mRNA expression of various 5-HT receptors (i.e., 5-HT1A, -1F, -4, and -5), which are capable of inducing greater 5-HT turnover. Moreover, six serotonin related genes had a treatment by day interaction whereby HS calves had greater expression of *TPH1*, *5-HT1B*, *-1D*, *-2C*, *-6*, and *-7* genes at d 1 and/or 21 relative to CL calves. These diverse patterns of 5-HT receptors expression have been previously reported in cerebral tissue from heat stressed mice at different ages [21], and in bovine immune cells with dissimilar 5-HT concentration levels [11]. Interestingly, the only 5-HT receptor that had greater mRNA expression in CL calf blood leukocytes was *5-HT2A*. This 5-HT receptor is expressed by platelets [48] and immune cells, including monocytes [49], dendritic cells [50], and T-cells [9]. Inoue *et al*. (2011) reported that 5-HT2A enhances interferon gamma (IFN- γ) and IL-2 production by T-cells [9], while others have reported that signaling through 5-HT2A enhances tumor necrosis factor alpha (TNFα) secretion [51], and inhibits TNFα induced IL-6 production [52]. Further, the responsiveness of 5-HT2A in the brain has been reported to increase after exposure to high environmental temperatures [53], and activation of the receptor at this site elicits hyperthermia [54]. Thus, considering the diverse functions of 5-HT receptors, it has yet to be determined whether their mRNA expression is translated to protein, altered by immune cell signaling, and/or changed by environmental temperatures.

Throughout the pre-weaning period, the immune cells of CL calves had greater blood leukocyte mRNA expression of the transcription factor *TBX21*, a master regulator of T-helper 1 cell development, and tended to have greater expression of the transcription factor *FoxP3*, which is important for T-regulatory (**T-reg**) cell development and function. The greater expression of these two master transcription factors in CL calves compared to HS calves may indicate that heat stress suppresses the development and/or differentiation of the bovine adaptive immune system. The concomitant increase of cytokine transforming growth factor beta 1 (*TGFB1*) mRNA expression, which accompanies the upregulation of *FoxP3*, further supports this hypothesis, as it favors the differentiation of naïve T-cells into Tregs [5]. Additionally, the toll-like receptor 4 (*TLR4*) was greater in the CL group. Toll-like receptors are expressed on innate immune cells, such as dendritic cells and macrophages, and function to recognize gram-positive (lipoteichoic acid; LTA) and gram-negative (lipopolysaccharide; LPS) bacteria through TLR2 and TLR4, respectively. Thus, it appears that providing heat stress abatement increases leukocyte’s pathogen recognition capacity in dairy calves. These results support the theory that heat stress hinders immune system development, and that providing heat stress abatement to dairy calves’ aids to ensure a more mature, differentiated and functional immune system at a younger age.

LPS stimulation increased peripheral blood leukocyte secretion of 5-HT in both treatments. However, CL calves secreted less 5-HT and more IL-6 in culture supernatant after the LPS-challenge relative to HS calves. Immune cells secrete IL-6 in response to LPS stimulation, which supports the growth and differentiation of T and B lymphocytes [5, 55, 56]. Interestingly, it has been reported that 5-HT attenuates IL-6 production by LPS-stimulated human whole blood [57]. Similar results have been reported for mice injected with LPS and 10 μg/μL of 5-HT [58]. These results may explain why our CL calves, which expressed less 5-HT than HS calves, had a greater capacity to secrete IL-6 after LPS-stimulation. Another contributing factor may be that CL calves were more responsive to LPS stimulation because they expressed greater *TLR4*, which is the toll-like receptor that recognizes LPS. Further research, probably using purified immune cell populations, is needed to determine the involvement of 5-HT in immune responses during heat stress in the bovine.

Peripheral leukocyte populations were assessed using flow cytometry at d 21 and 47 of age. Although no differences were found in peripheral leukocyte populations such as granulocyte and monocyte percentages between treatments, CL calves had a substantially higher frequency of B-cells than HS calves. Greater postnatal peripheral blood mononuclear cell proliferation has been previously reported in prenatal CL relative to prenatal HS calves [3, 36]. Considering that immune cell proliferation accompanies immune cell activation, the expression of cell-adhesion/activation molecules was analyzed to characterize immune cell activation. It has been reported that upon activation, leukocytes downregulate CD62L+ expression and increase CD44+ expression [5, 59]. Calves that experienced pre- and postnatal heat stress had greater CD44+ expression on granulocytes, monocytes, and lymphocytes populations, suggestive of greater activation. The increase in CD44+ expression has been previously reported on activated macrophages [60] and T-cells in the bovine [59] and it is typically used to recognize effector and memory T-cells in rodent and human immune cells [5, 59]. Further, HS calves had lower CD62L+ MFI expression in granulocytes at d 47, suggestive of a more activated innate immune system rather than the adaptive immune system [61].

## Conclusions

Dairy calves exposed to chronic heat stress during the pre- and postnatal developmental phases had impaired IgG production, reduced RBC counts, and reduced gene expression of *HSP70*, *TBX21*, *Foxp3*, *TGFB1*, and *TLR4* relative to CL calves. Additionally, chronic heat stressed calves had reduced circulating 5-HT concentrations during the preweaning phase, which in turn, might alter 5-HT-related gene expression in peripheral blood leukocytes. Our work provides evidence that early life heat stress abatement could promote peripheral 5-HT metabolism and support the development of humoral immune responses in dairy calves, which could ultimately aid in disease resilience.

## Supporting information

S1 Fig Gating strategies to distinguish T cells, and monocytes subsets, and their activation markers. Representative gating strategy to distinguish (**A**) granulocytes, CD4+ and CD8+ T-cell populations, and their expression of CD44 and CD62L, and (**B**) CD14+ monocytes, TCRγδ+ T-cells, and CD21+ B-cells. The CD4 and CD8 T-cell subsets were identified by sequentially gating on single cells (singlets), the lymphocyte population, CD4+ and CD8+ positive subpopulations, and their CD62L and CD44 subpopulation, whereas TCRγδ+ T-cells and CD21+ B-cells were identified by sequentially gating on live (propidium iodide (**PI**) negative cells), the lymphocyte population, and either TCRγδ+ or CD21+ populations, while the CD14+ monocyte population was gated from singlets. FSC, forward scatter; SSC, side scatter; A, signal area; H, signal height.
(TIFF)

S2 Fig Treatment by day interaction of differentially expressed genes of peripheral blood leukocytes isolated from dairy calves at 1, 21 and 42 days of age. mRNA expression of peripheral blood leukocytes isolated from dairy calves at 1, 21 and 42 days of age. Dairy calves were exposed to heat stress (HS; n = 6) or heat stress abatement (CL; n = 6) across pre- and postnatal phases (late gestation and pre-weaning) for a total of 102 d. Elevated average ΔCt is indicative of smaller mRNA expression. Blue and red bars denote dairy calves’ postnatal CL and HS treatments, respectively. Asterisks indicate significance (*P* ≤ 0.05).
(TIFF)

S1 Table Gene names and accession number, sequence and source of primers used for gene expression analysis. ^1^Primer sequences were obtained from cited sources or were designed using Primer3 software (Input v.0.4.0) with sequences obtained from GenBank (http://www.ncbi.nlm.nih.gov/). Primer sequences are presented as 5’ to 3’ (forward) and 3’ to 5’ (reverse). All primer pairs displayed melting curves with a single peak, indicative of a pure, single amplicon, confirmed the specificity of the primer pair.
(XLSX)

S2 Table Back transformed serotonin and IL-6 concentrations. ^a^ Calves were exposed to heat stress (HS; n = 6) or heat stress abatement (CL; n = 6) treatments (Trt) across pre- and postnatal periods (late gestation and pre-weaning) for a total of 102 d. ^b^ Serotonin and interleukin-6 concentrations data were log-transformed for statistical analysis due to lack of normality, and least squares means were backtransformed using the formula exp(log(LSM)) and the standard error (SEM) was backtransformed using the exp(log(LSM)*log(SEM).
(XLSX)

S1 Data (XLS)
