# Supplementary figures and images for: Chronic heat stress delays immune system development and alters serotonin signaling in pre-weaned dairy calves
Source: PLoS One. 2021 Jun 4;16(6):e0252474. doi: 10.1371/journal.pone.0252474 (PMC8177632; doi:10.1371/journal.pone.0252474)

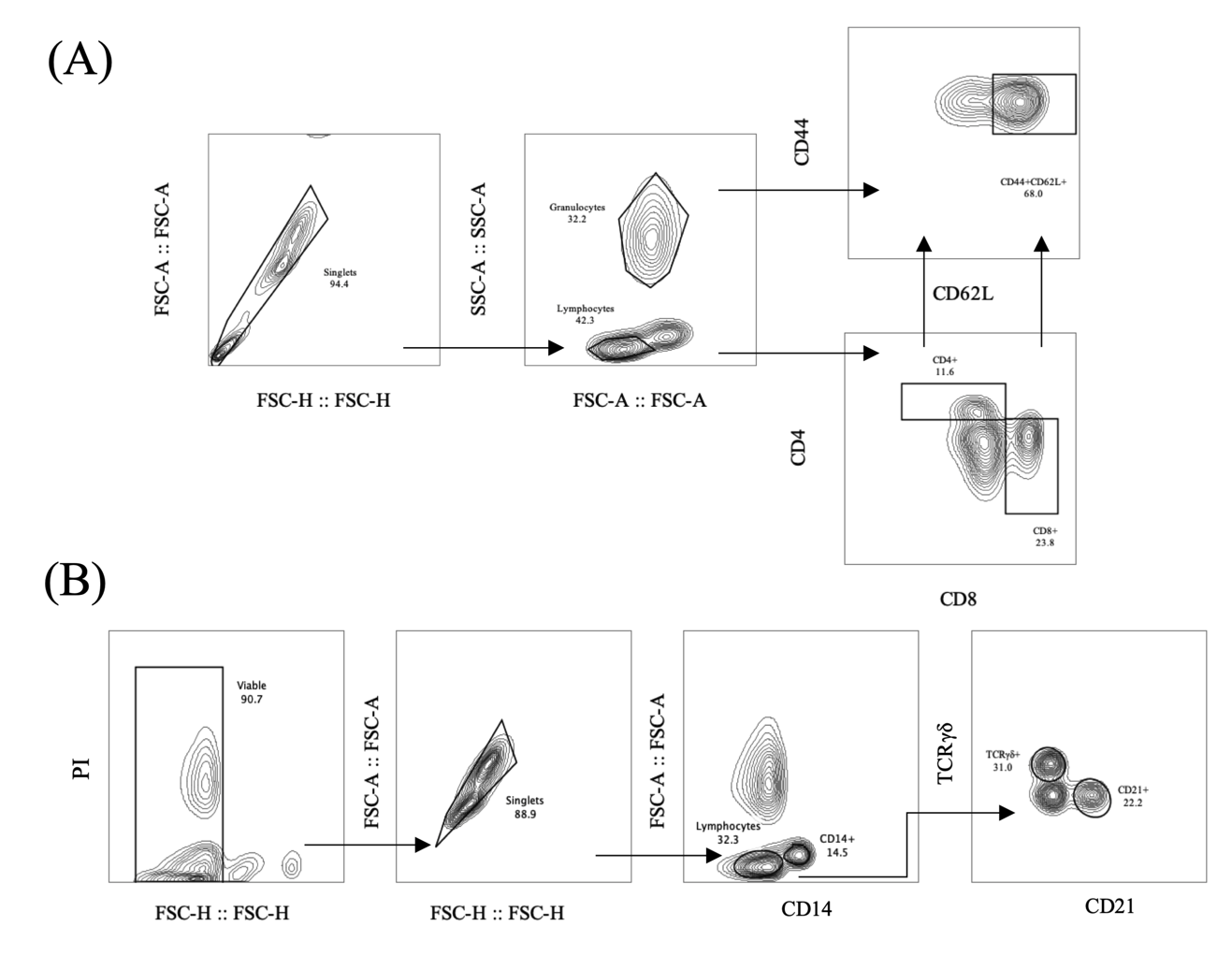

Supplement: S1 Fig — Representative gating strategy to distinguish (A) granulocytes, CD4+ and CD8+ T-cell populations, and their expression of CD44 and CD62L, and (B) CD14+ monocytes, TCRγδ+ T-cells, and CD21+ B-cells. The CD4 and CD8 T-cell subsets were identified by sequentially gating on single cells (singlets), the lymphocyte population, CD4+ and CD8+ positive subpopulations, and their CD62L and CD44 subpopulation, whereas TCRγδ+ T-cells and CD21+ B-cells were identified by sequentially gating on live (propidium iodide (PI) negative cells), the lymphocyte population, and either TCRγδ+ or CD21+ populations, while the CD14+ monocyte population was gated from singlets. FSC, forward scatter; SSC, side scatter; A, signal area; H, signal height. (TIFF) [file pone.0252474.s001.tiff]

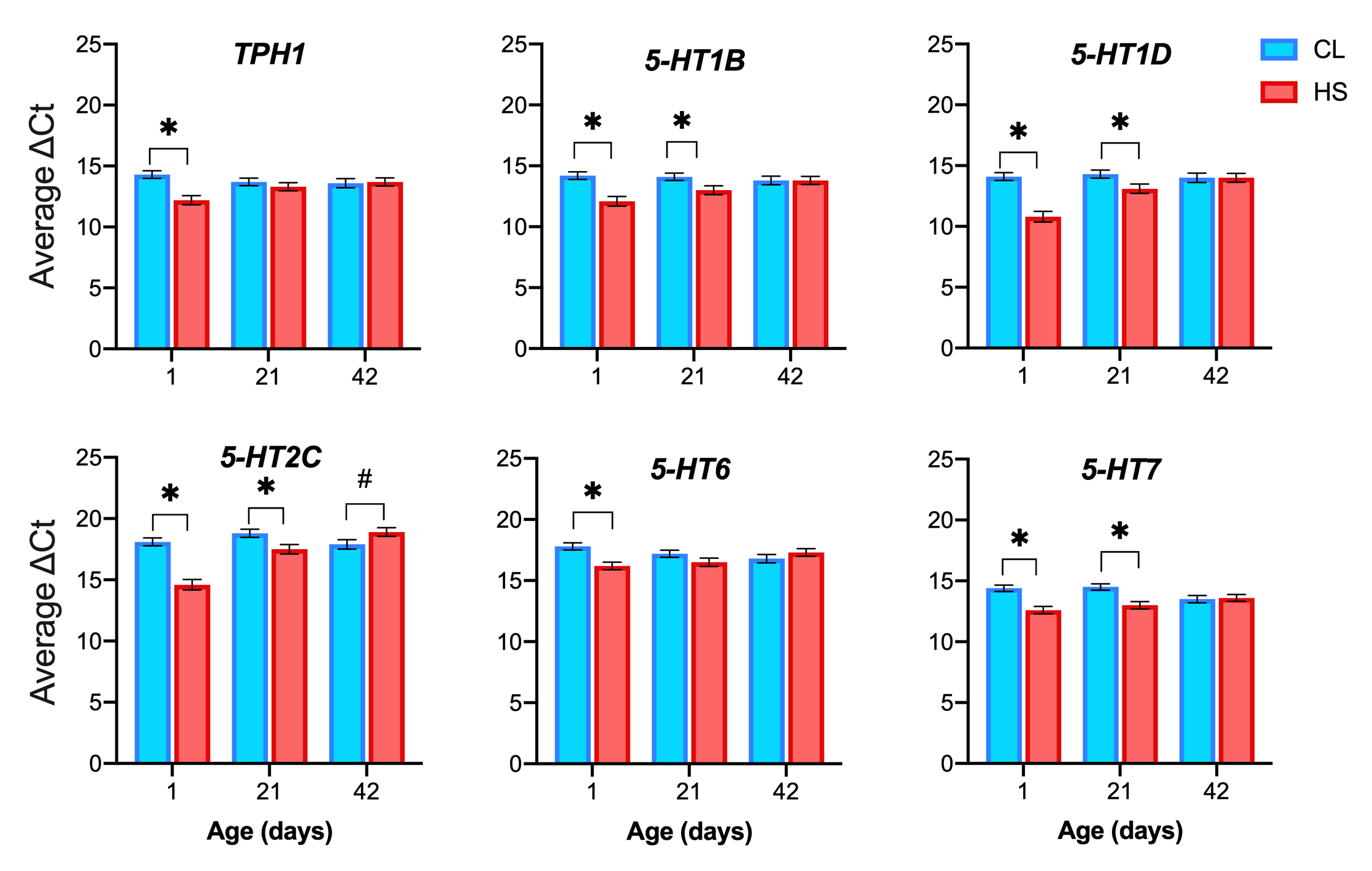

Supplement: S2 Fig — mRNA expression of peripheral blood leukocytes isolated from dairy calves at 1, 21 and 42 days of age. Dairy calves were exposed to heat stress (HS; n = 6) or heat stress abatement (CL; n = 6) across pre- and postnatal phases (late gestation and pre-weaning) for a total of 102 d. Elevated average ΔCt is indicative of smaller mRNA expression. Blue and red bars denote dairy calves’ postnatal CL and HS treatments, respectively. Asterisks indicate significance (P ≤ 0.05). (TIFF) [file pone.0252474.s002.tiff]
